# Multidisciplinary Approach to Hepatic Metastases of Intracranial Hemangiopericytoma: A Case Report and Review of the Literature

**DOI:** 10.1155/2015/214306

**Published:** 2015-05-18

**Authors:** Dimitrios K. Manatakis, Spiridon G. Delis, Nikolaos Ptohis, Penelope Korkolopoulou, Christos Dervenis

**Affiliations:** ^1^Surgical Department, “Konstantopouleio” General Hospital, Nea Ionia, 14233 Athens, Greece; ^2^Interventional Radiology Department, “G. Gennimatas” General Hospital, 11527 Athens, Greece; ^3^1st Department of Pathology, Medical School, National and Kapodistrian University of Athens, 11527 Athens, Greece

## Abstract

Hemangiopericytoma is a rare primary tumor originating from Zimmerman's pericytes, with significant metastatic potential. Hepatic metastatic disease requires an aggressive approach by a multidisciplinary team of dedicated oncology specialists, to prolong survival in selected patients. We report on a patient with recurrent hepatic metastases of grade II intracranial hemangiopericytoma 5 years after initial treatment, managed by a stepwise combination of liver resection, radiofrequency ablation, and transarterial embolization. Although metastatic disease implies hematogenous dissemination, long-term survival after liver resection has been reported and major hepatectomies are justified in patients with adequate local control. Liver resections combined with transarterial embolization are highly recommended, due to hypervascularity of the tumor.

## 1. Introduction

Noncolorectal, nonneuroendocrine hepatic metastases are a diverse group of secondary liver neoplasias, exhibiting variable clinical behavior and characteristics. Particularly in cases of rare primary tumors, where limited datasets are available and no official guidelines are published, a multidisciplinary team (MDT) approach is mandated, to provide comprehensive assessment, consultation, and treatment.

We present our experience with a case of recurrent hepatic metastases from an intracranial hemangiopericytoma (HPC), managed by an MDT of oncologic surgeons, medical oncologists, interventional radiologists, and dedicated pathologists.

## 2. Case Presentation

**Figure 1:**
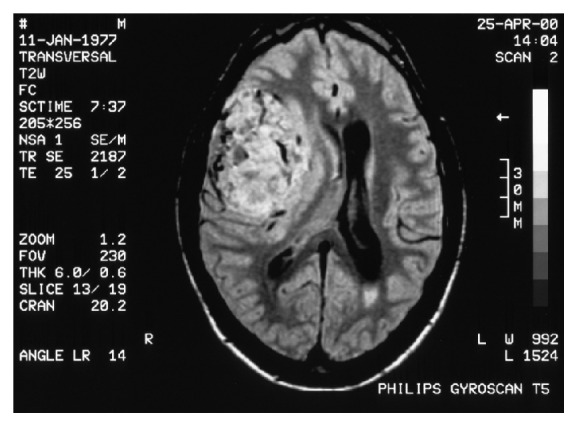
Brain primary tumor.

A 23-year-old Caucasian male underwent a right temporal lobe glomus tumor excision. Seven years later he was diagnosed with local recurrence and underwent two craniotomies to achieve radical resection (Figure 1). Histopathology revealed an HPC grade II. No adjuvant chemoradiotherapy was instituted.

**Figure 2:**
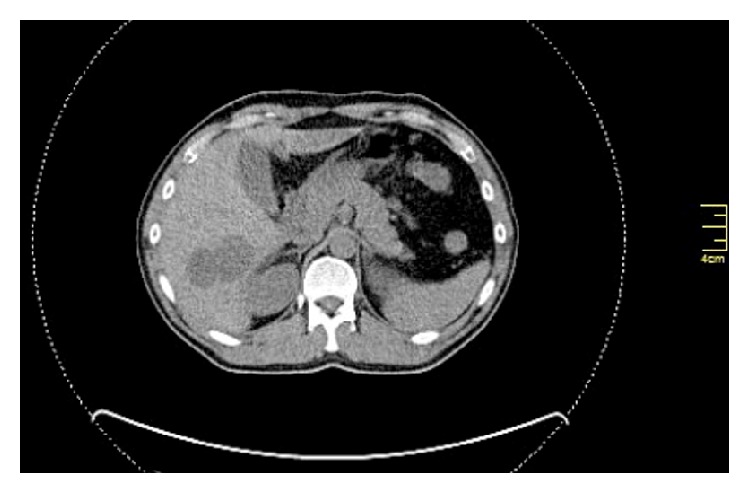
Liver metastases, segment VI.

**Figure 3:**
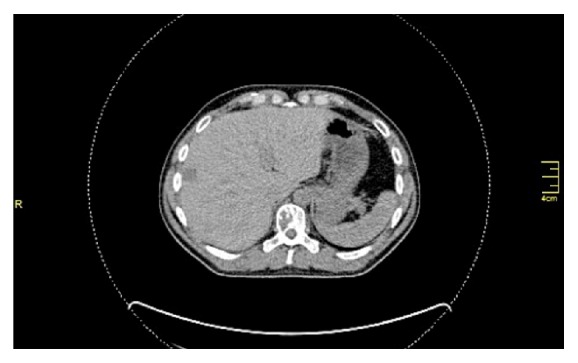
Liver metastasis, segment VIII.

Five years after the last intervention, he was admitted to hospital due to diverticulitis and an incidental finding of a small lesion in the body of T10 vertebra was demonstrated on CT scans, as well as multiple, atypical, hypervascular lesions in both liver lobes, the larger being 8 cm in diameter, initially considered hemangiomas (Figures 2 and 3). Magnetic resonance imaging, however, was consistent with metastatic HPC lesions rather than benign hemangiomas. Although positron emission tomography (PET) revealed strong uptake of the contrast medium in the T10 lesion, no uptake in the liver was documented. Tumor markers (CEA, *α*FP, and Ca19-9) were within normal limits.

**Figure 4:**
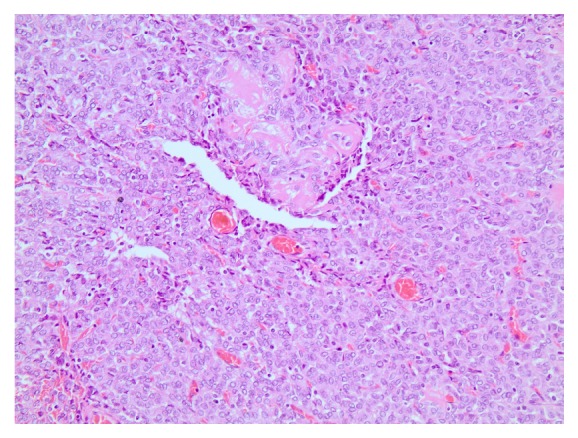
H&E stain, 200x.

**Figure 5:**
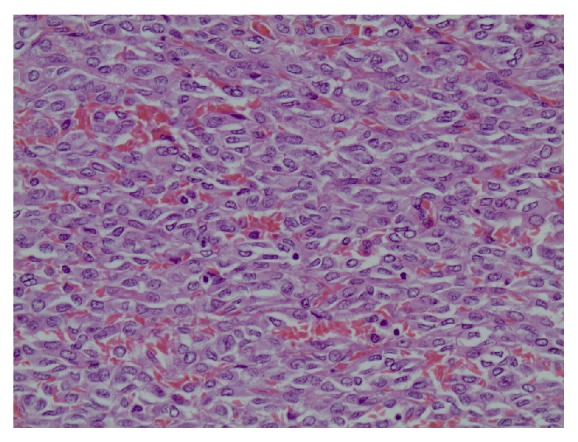
H&E stain, 400x.

**Figure 6:**
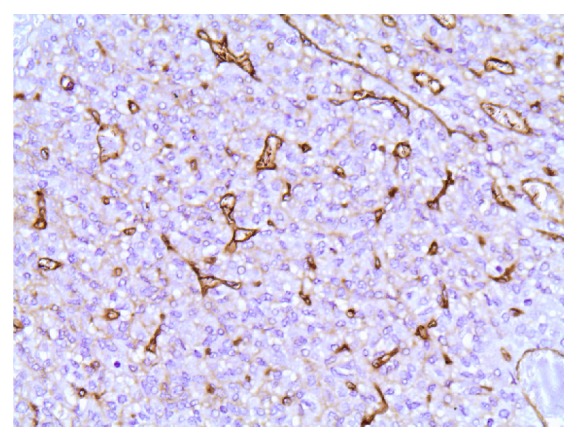
CD34 stain, 40x.

A right hepatectomy plus radiofrequency ablation of one lesion in the left lobe was decided and the patient had an uneventful postoperative course. Histopathology revealed two tumors (8.7 cm and 3.8 cm) consisting of relatively bland mesenchymal cells, with no unique characteristics, packed around an elaborate network of vessels (Figures 4 and 5), confirming the hypervascularity and nonepithelial origin of the neoplasms, which exhibited mild nuclear pleomorphism, low mitotic rate (<5 mitoses/10 HPF), and few areas of necrosis. Immunohistochemistry was positive for CD34 (Figure 6) and vimentin and negative for S100. Stain for EMA was focally positive. The immunoreactivity profile was identical to the previous report of the primary brain tumor.

**Figure 7:**
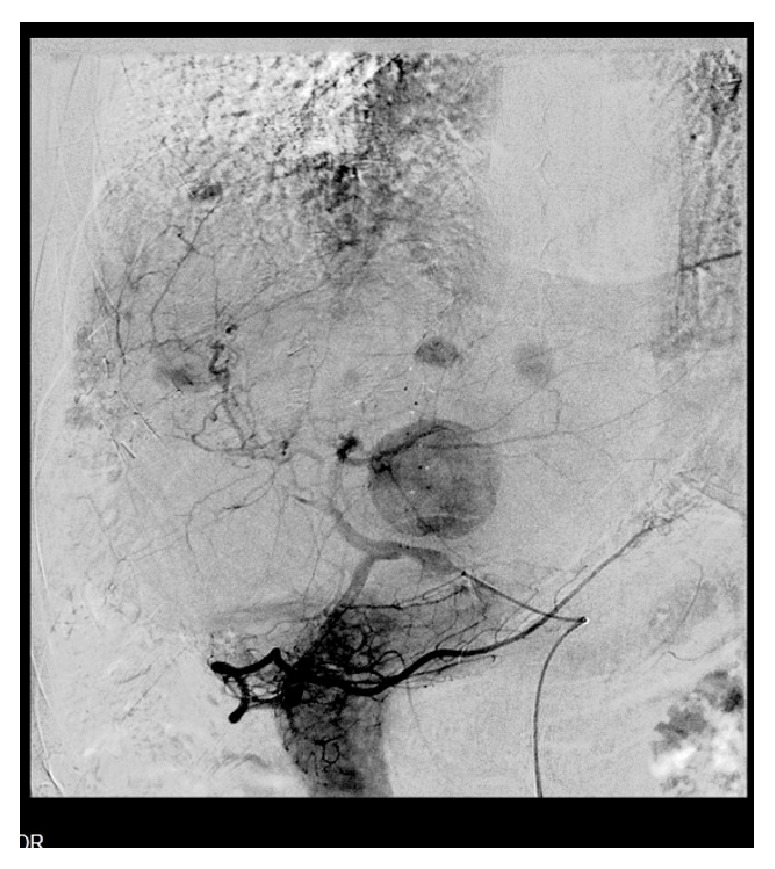
Multiple hypervascular hepatic lesions.

**Figure 8:**
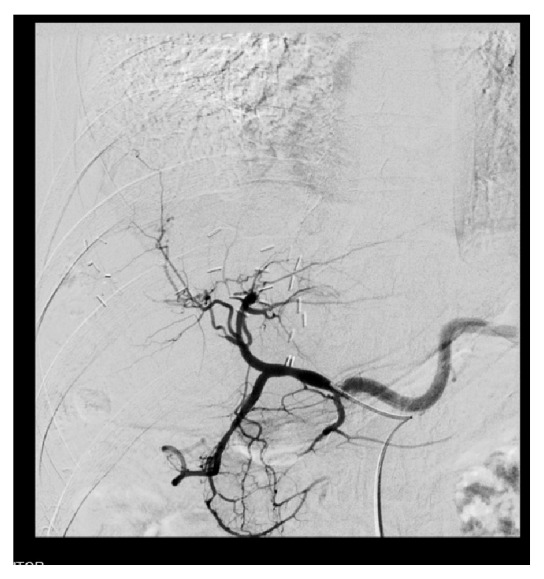
Disappearance of hepatic lesions after transarterial embolization.

One year after liver resection, follow-up CT scan revealed no local recurrence in the brain cavity, but three metastatic lesions in the liver parenchyma (segments II and IV) and slow progression of the T10 lesion. The MDT proposed stereotactic radiosurgery for the bone metastasis and elective transarterial embolization (TAE) of the liver lesions, as bridging therapy to a second hepatectomy. Two of the liver lesions were hypervascular, although the caudate lobe metastasis was only slightly vascular with a capillary network. Transarterial embolization was successful, due to hyperselective arterial catheterization (Figures 7 and 8).

## 3. Discussion

Hemangiopericytoma is a rare mesenchymal tumor, originating from Zimmerman's pericytes, which are contractile spindle cells, surrounding capillaries and postcapillary venules, and regulate capillary flow and permeability [1]. Most common anatomic locations involved are lower extremities, retroperitoneum, and meninges, with both genders equally affected [1, 2]. Intracranial HPCs constitute less than 1% of all intracranial tumors [3].

Generally HPCs are aggressive tumors, with 5-year local recurrence rate of 65% and 5-year distant metastasis rate of 33%, with lung, liver, and bones being the most frequent metastatic locations [3].

Clinical symptoms are vague and nonspecific, depending on tumor location and size [4]. Pain is usually a late presentation, while hypoglycemia represents a unique endocrine paraneoplastic syndrome, caused by oversecretion of insulin-like growth factor II [5].

Hypervascularity is the prominent imaging characteristic and preoperative differential diagnosis from other vascular lesions is necessary, due to differences in treatment and prognosis. Unenhanced liver ultrasound reveals hypoechoic lesions, which become markedly hyperechoic during the arterial phase of contrast medium administration [6]. On CT scans, HPCs exhibit arterial phase enhancement and usually have well-defined borders [6].

Magnetic resonance imaging reveals well-defined, lobular lesions, with an isointense signal in T1WI and slightly long signal in T2WI unenhanced scans. Following gadolinium administration, a heterogeneous enhancement is observed, due to cystic degeneration, focal tumor necrosis, or flow voids [4, 6].

Unlike meningioma, which has a hyperplastic effect on adjacent bone, HPCs are shown to exert an osteolytic effect. Moreover, no dural tail sign is observed [4, 6].

Fluorodeoxyglucose (FDG) PET may be helpful in revealing multiple distant metastases. As in our case, HPCs show avid enhancement on CT, but low FDG uptake on PET, indicative of low glucose metabolism [7].

Accurate preoperative diagnosis may still be challenging, despite high-tech imaging modalities, and histopathology and immunohistochemistry confirm the diagnosis. Hemangiopericytomas are highly cellular and vascularized tumors, consisting of tightly packed, round to fusiform cells, around a well-developed, elaborate branching “staghorn” vasculature [1, 8].

According to the World Health Organization classification, HPCs are graded as being differentiated (grade II) and anaplastic (grade III) [3]. Signs of anaplasia include high mitotic rate (>5 mitoses per HPF) and/or necrosis, plus at least two of the following features: hemorrhage, moderate to high nuclear atypia, and cellularity [8].

Immunohistochemically tumor cells usually express CD34 and vimentin but not EMA and S100 [1, 3]. Immunoreactivity patterns generally vary among HPCs and no antibody is 100% sensitive or specific [3]. However immunoprofiles are helpful, to exclude meningiomas and solid fibrous tumors [3, 4].

Despite advances in diagnostic and therapeutic modalities, management of HPCs remains challenging and problematic. Due to the tumor's rarity, most published studies are retrospective cohorts with small numbers of patients. Consequently, there are no concrete treatment recommendations based on level I evidence.

Surgery is the mainstay of treatment. Indeed, all authors agree that gross total resection increases overall survival (OS) and recurrence-free interval [3, 9–12]. However, hypervascularity of the tumor and close relation to delicate intracranial structures make complete resection of brain primaries possible in only 50–60% of patients [3, 11]. As for hepatic metastatic disease, there are no guidelines on resection margins and intraoperative ultrasonography may be helpful to achieve R0 resections. Liver transplantation has been reported in a case of refractory hypoglycemia [13].

The role of adjuvant radiotherapy is controversial. Safe conclusions cannot be drawn, due to small sample sizes and retrospective character of available studies. Zweckberger et al. propose radiotherapy to grade II patients with subtotal resections and to all grade III patients [3]. Schiariti et al. found that radiation reduces risk of local recurrence but does not prolong recurrence interval [9]. On the other hand, Rutkowski et al. found no benefit from gross total resection plus radiotherapy when compared to gross total resection alone, and total doses of >50 Gy were associated with increased mortality rates. Similarly, in cases of subtotal resection, addition of radiation offered no survival benefit [10].

Stereotactic radiosurgery (gamma-knife, cyber-knife) has been used in cases of recurrent or residual disease, with promising results [3]. Ecker et al. reported a 93% response rate, with 42 out of 45 tumors obliterated, decreased, or controlled [11]. Veeravagu et al. reported that stereotactic radiosurgery increases time to recurrence and OS [14]. However it does not decrease the risk for distant metastases, which are a cause of significant morbidity and mortality.

Systemic chemotherapy has shown only disappointing results and its role is purely palliative [11, 12]. On the other hand, there is a growing body of evidence on the value of antiangiogenic drugs (sunitinib, sorafenib, pazopanib, bevacizumab/temozolomide, and endostatin/ginsenoside Rg3). The rationale behind their use is the expression of platelet-derived growth factor receptor (PDGFR) and vascular endothelial growth factor receptor (VEGFR) by the HPC tumor cells. Small experimental trials or case reports have been published to date, reporting partial response or stable disease course for several months; however, these results need validation in larger controlled studies [2, 5, 15–17].

Although HPC has been described as borderline malignant, its clinical behavior is difficult to predict and long-term follow-up is indicated, since recurrence and metastasis have been reported even after prolonged disease-free intervals [1, 9, 18]. Factors affecting prognosis are mainly tumor grade and completeness of resection; however, in multivariate analysis, they do not reach statistical significance in most papers due to small sample sizes [9].

Comparison between low- and high-grade tumors has shown that even grade II tumors have significant metastatic potential and often relapse [3]. High-grade tumors recur earlier than low-grade tumors and decrease OS rates [9, 11].

The survival benefit of gross total resection applies to both CNS and extra-CNS HPCs, although extra-CNS tumors tend to be larger and more advanced and with shorter OS [12].

In their study, Damodaran et al. reported OS at 5, 10, 15, and 20 years at 79%, 56%, 44%, and 22%, respectively [19]. For grade II tumors, OS was 216 months, while for grade III tumors OS was 142 months. Local recurrence rates at 5, 10, and 15 years were 20%, 54%, and 77%, while distant metastasis rates at 5, 10, and 15 years were 10%, 31%, and 77%.

Although liver metastatic disease is a surrogate marker of tumor hematogenous dissemination, long-term survival after hepatectomy is reported. Extrahepatic disease is not a contraindication to liver resection in case of local control, as reported in our case. In essence, if the time interval between the primary lesion and the liver metastatic disease is prolonged, major hepatectomy is justified in young individuals. In case of liver recurrence, re-resection combined with TAE is highly recommended, due to the hypervascular nature of the tumor [20].
